# Adult‐Onset Partial Unilateral Lentiginosis: A Rare Pigmentary Mosaicism With Clinical Response to Tranexamic Acid

**DOI:** 10.1002/ccr3.71113

**Published:** 2025-10-02

**Authors:** Niranjan Pudasaini, Sabin Poudel, Aashutosh Pokharel, Anup Mishra, Anil Kumar Singh Dangol, Manisha Chapagain

**Affiliations:** ^1^ Department of Dermatology Shree Birendra Hospital Kathmandu Nepal; ^2^ Shree Birendra Hospital Kathmandu Nepal; ^3^ Nepalese Army Institute of Health Sciences Kathmandu Nepal

**Keywords:** histopathology, lentiginosis, mosaicism, tranexamic acid

## Abstract

Partial unilateral lentiginosis (PUL) is a rare pigmentary disorder characterized by numerous lentigines grouped within normal skin in a segmental pattern with sharp demarcation at the midline. The authors hereby present a case of a 40‐year‐old female, who presented with multiple asymptomatic, gradually progressive, brownish flat skin lesions on her left breast, abdomen, and left half of the trunk for 16 years. Diagnosis was made clinically via properly documented history, physical examination, and histopathological evaluation. Although treatment is unnecessary, many patients seek consultations for cosmetic reasons. Our patient is showing a good response to the prescribed tablet tranexamic acid and topical kojic acid cream, along with regular application of sun protection cream SPF 50+. This is a rare case of adult‐onset PUL showing good clinical improvement with oral tranexamic acid combined with kojic acid cream.

**Abbreviations**
mg: milligram
NF1: neurofibromatosis type 1
PUL: partial unilateral lentiginosis

**Summary**

- Partial unilateral lentiginosis (PUL) is a rare pigmentary mosaicism that requires careful clinical and histopathological evaluation to differentiate from other segmental lentiginoses.
- This case highlights the potential benefit of oral tranexamic acid and topical kojic acid as a well‐tolerated and cosmetically effective treatment option in adult‐onset PUL.

## 1 Introduction

Partial unilateral lentiginosis (PUL) is a rare benign pigmentary disorder where multiple lentigines are typically found clustered together within an area of normal skin [1]. The lesions are confined to one or more dermatomes in a segmental pattern with a sharp demarcation at the midline [2, 3]. In most patients, the lesions first appear or are often noticed during early childhood [4]. It can occur anywhere on the body, including the face, neck, trunk, and extremities [3]. It is believed to occur due to somatic mosaicism of several “developmental” pigmentary genes confined to the neural crest melanoblast [5, 6]. Due to its similarity with nevus spilus macules and other pigmentary disorders, it needs to be carefully diagnosed with proper history, dermatological examination, and histopathological evaluation [6]. Although treatment is not necessary, people tend to seek consultation due to cosmetic reasons [7]. There is no established standard treatment for PUL. However, available treatment modalities include laser therapy and topical agents such as tretinoin and hydroquinone [8]. This case report has been reported in line with CARE guidelines [9].

## 2 Case History

**FIGURE 1:**
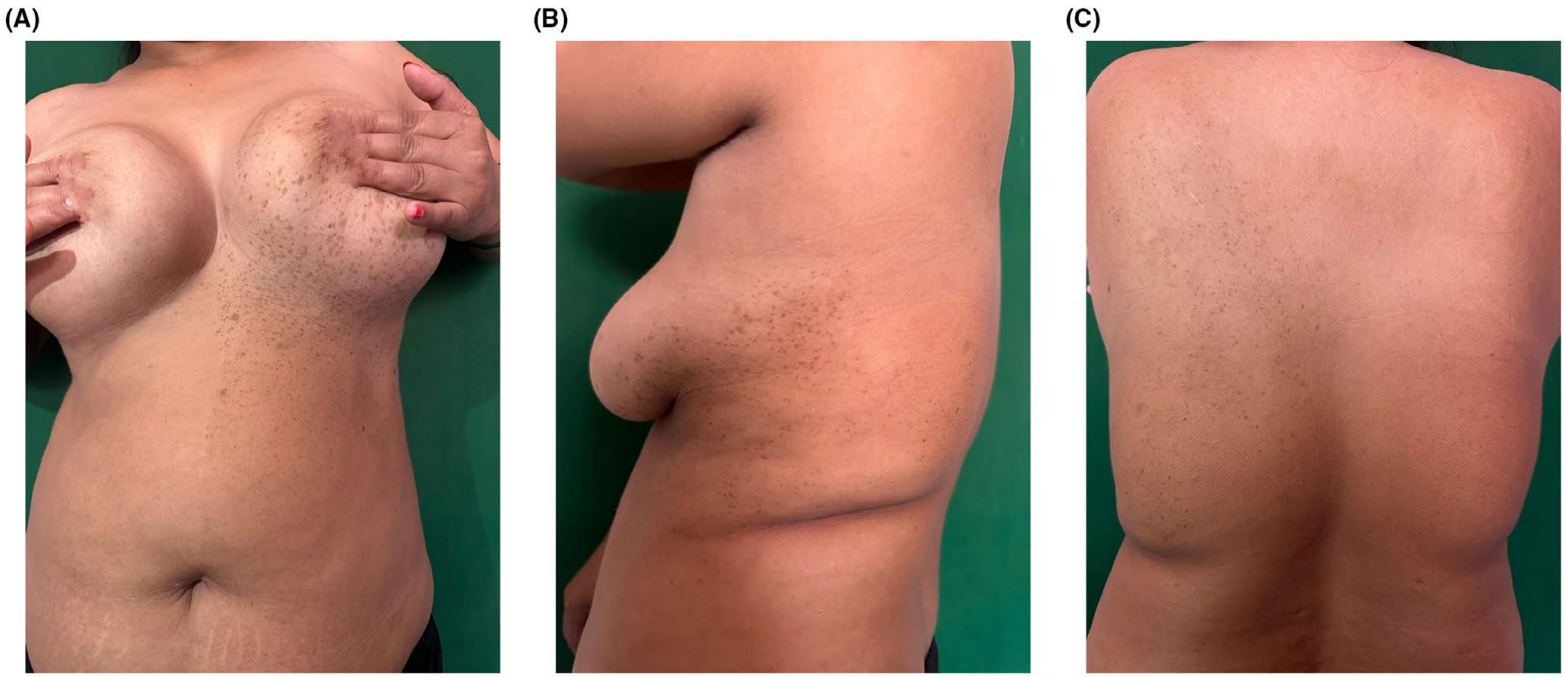
(A) Anterior view showing multiple unilateral brownish flat skin lesions extending from the left breast to the upper part of the abdomen on the left side of the body. (B) Lateral view showing multiple unilateral brownish flat skin lesions on the left side of the body, located infra axillary. (C) Posterior view showing multiple unilateral brownish flat skin lesions on the left side of the trunk.

A 40‐year‐old female, farmer by occupation with no known comorbidities, presented to the dermatology department with multiple asymptomatic, gradually progressive, brownish flat skin lesions on her left breast, abdomen, and left half of the trunk for 16 years, as shown in Figure 1A–C. The patient reported no history of congenital pigmentation, prior inflammatory skin conditions, or trauma at the sites of discoloration.

**FIGURE 2:**
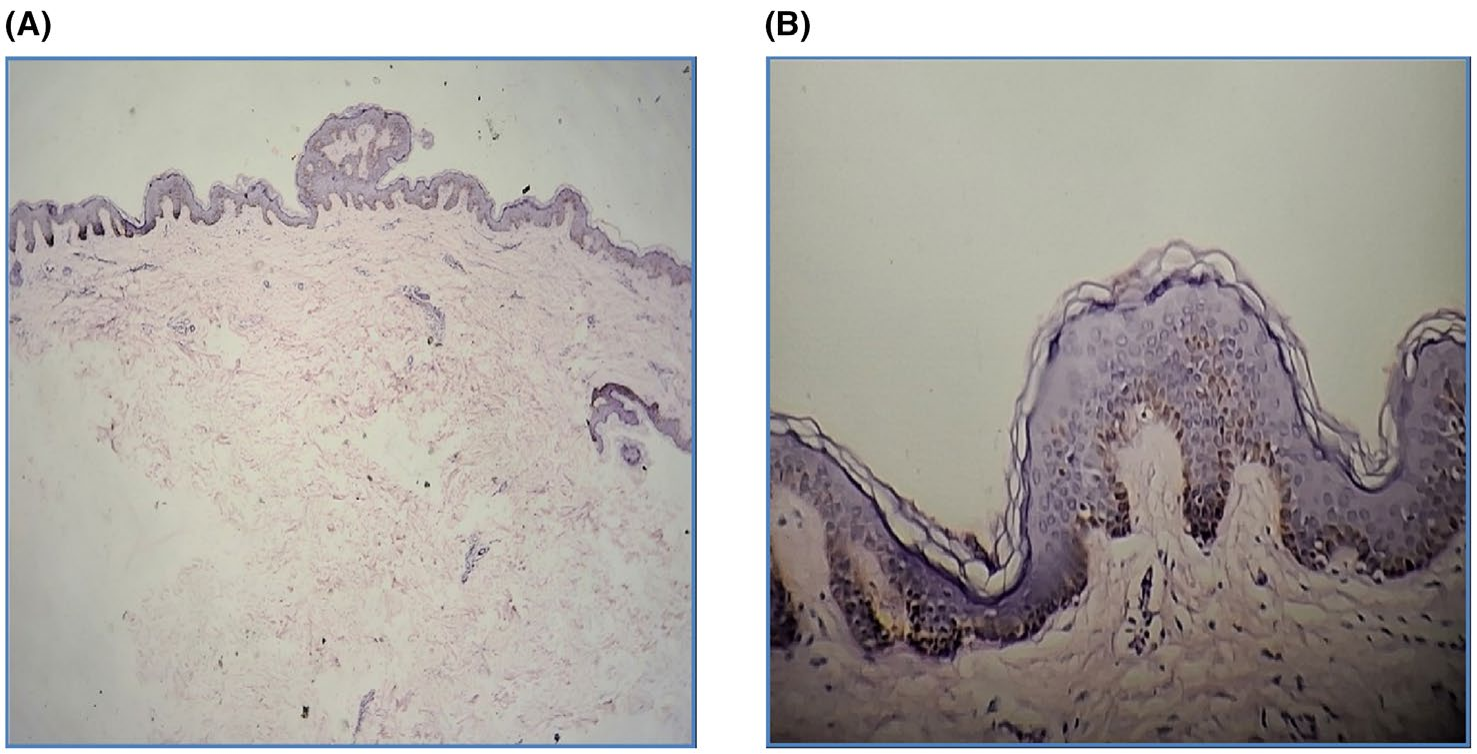
(A) 4× view showing an increase in melanin pigmentation in the basal layer of the epidermis. (B) 40× view showing an increase in melanin pigmentation in the basal layer of the epidermis.

On dermatoscopic examination, there was involvement of the left breast and upper abdomen extending to the infra‐axillary region and back in the form of multiple brownish macules ranging from 1 mm × 1 mm to 1 cm × 1.2 cm in a discrete to confluent manner. These lesions were sharply demarcated at the midline over normal skin. She had no axillary freckling or any other manner suggestive of neurofibroma. Results of routine laboratory examinations, including complete blood cell count, liver and kidney function tests, ECG, and serological profile, were within normal limits. Ophthalmic and neurological evaluations were conducted to rule out associated neurocutaneous syndromes such as neurofibromatosis type 1, LEOPARD syndrome, and segmental neurofibromatosis. Histopathological examination of a biopsy specimen from the lesion showed increased melanin pigment in the stratum basale, with mild inflammatory infiltrates comprising lymphocytes in the dermis as shown in Figure 2A,B, Confirming, confirming the diagnosis of partial unilateral lentiginosis. There was no evidence of dysplasia or malignancy. She was prescribed tablet tranexamic acid 500 mg once a day and topical kojic acid cream applied locally at bedtime. Counseling was done on the necessity of avoiding sun exposure, the use of long‐sleeve sun protective clothes, and regular application of sun protection cream. She was kept on regular follow‐up, and the lesions were closely monitored.

## 3 Discussion

Lentigines are discrete, irregular, hyperpigmented macules caused by ultraviolet light exposure [10]. Lentigines that are arranged in a segmental pattern and do not cross the midline are referred to as PUL [11]. The lesions commonly occur on the head and neck, followed by the trunk and extremities [4]. The patient in our case had localized lesions present on her left breast, left half of the trunk, and abdomen.

The population incidence of PUL is unknown, but it is generally believed to be uncommon due to the very small number of reported cases in the literature. Although its exact pathogenesis is not fully discovered, it is thought to be due to somatic mosaicism, where a postzygotic mutation occurs in melanoblasts derived from the neural crest during embryonic development [12]. The pattern of distribution in PUL is characteristically segmental, respecting the midline and often following dermatomal or Blaschko lines. This unique distribution further supports the underlying theory of mosaicism. In this case, the segmental arrangement involving the left half of the trunk and abdomen was consistent with classical PUL presentation.

PUL is typically a benign and isolated pigmentary disorder with minimal or no associated systemic symptoms. However, rare associations such as ocular involvement, neurofibromatosis type 1 (NF1), nevus depigmentosus, mental retardation, cerebrovascular anomalies, and focal epilepsy have been reported [2, 13]. In our patient, no such systemic or ocular associations were identified upon detailed ophthalmologic and neurologic evaluation.

It is crucial to distinguish PUL from other pigmentary conditions, particularly those with potential systemic implications or malignant transformation risks. The primary differential diagnosis includes neurofibromatosis type 1 (NF1), Peutz–Jeghers syndrome, LEOPARD syndrome, Nevus spilus, and segmental neurofibromatosis NF type 2. Among these, nevus spilus maculosa deserves special attention as it carries a potential risk of malignant transformation into melanoma. Hence, histopathological examination is essential, along with detailed history and clinical examination for accurate diagnosis. Histopathological findings commonly include basal hyperpigmentation, dermal melanophages, and perivascular lymphocytic infiltration [4]. In our case, basal hyperpigmentation and perivascular lymphocytic infiltration were present, supporting the diagnosis of PUL.

As this condition is benign and harmless, treatment is considered only for the improvement of the patient's cosmetic appearance. Different treatment options available are laser therapy and topical agents such as tretinoin and hydroquinone [8]. However these options are often costly and have variable outcomes [7], due to which, in our case, tranexamic acid and kojic acid were prescribed along with sun protection cream. Tranexamic acid, along with kojic acid, inhibits melanogenesis and also shows anti‐angiogenic, anti‐inflammatory, and skin barrier repairing action [14]. As chronic exposures to UV light are a primary etiological factor [10], the patient was counseled regarding strict photoprotective measures such as avoidance of sunlight exposure and use of long sleeves.

The long‐term prognosis of PUL is unknown [15] as data on progression or complications are limited due to its rarity. Continuous follow‐up is advised to monitor potential changes or the development of new associated features. The patient presented here is on close follow‐up; lesions are being closely monitored, and she is showing a good response.

## 4 Conclusion

To the best of our knowledge, this is the first documented use of tranexamic acid in the management of PUL. The patient demonstrated significant clinical improvement with a visible reduction in pigmentation during follow‐up without any side effects. The treatment was well tolerated and was cosmetically beneficial. These findings highlight tranexamic acid as a potential therapeutic option for the treatment of PUL.

## Author Contributions

**Niranjan Pudasaini:** conceptualization, supervision, writing – original draft, writing – review and editing. **Sabin Poudel:** writing – original draft, writing – review and editing. **Aashutosh Pokharel:** writing – original draft, writing – review and editing. **Anup Mishra:** writing – original draft, writing – review and editing. **Anil Kumar Singh Dangol:** conceptualization, supervision, writing – original draft, writing – review and editing. **Manisha Chapagain:** writing – original draft, writing – review and editing.

## Consent

Authors have obtained informed written patient consent for the use of their photographs and medical information to be published online and with the understanding that this information may be publicly available and discoverable via search engines. This consent form is not provided to the Editorial Office but has been retained by the author and will be provided whenever requested.

## Conflicts of Interest

The authors declare no conflicts of interest.

## Data Availability

The data supporting the findings of this case report, including de‐identified laboratory results and clinical images, are available from the corresponding author upon reasonable request.
